# Application of the entropy-DEMATEL-VIKOR multicriteria decision-making method in public charging infrastructure

**DOI:** 10.1371/journal.pone.0258209

**Published:** 2021-10-21

**Authors:** Hua Dong, Kun Yang

**Affiliations:** School of Economics and Management, North China Electric Power University Beijing, Beijing, China; Gonbad Kavous University, ISLAMIC REPUBLIC OF IRAN

## Abstract

As an energy-saving and environmentally friendly means of transportation, electric vehicles have been advocated and promoted by various countries, resulting in an increase in the number of electric vehicles. The improvement of public charging infrastructure not only drives the development of the electric vehicle industry but also solves the problems of user difficulty in charging and the low utilization rate of charging piles. From the perspective of electric vehicle (EV) user experience, this research establishes a framework of indicators, including the reputation level, service quality, convenience, economy and safety. Second, the objective entropy weight method and the subjective decision-making trial and evaluation laboratory (DEMATEL) method are combined to weight the indicators. Among the indicators, the comprehensive weights of market share (C2), app operation interface (C3), and charging mode (C5) are 0.107, 0.088, and 0.090, respectively, ranking in the top three. These three indicators should be given more attention by public charging infrastructure operators. Finally, three alternative public charging infrastructures are sorted by using the VlseKriterijuska Optimizacija I Komoromisno Resenje (VIKOR) method. Since the positive ideal solution Si of h1 (state grid) is 0.084, the negative ideal solution Ri is 0.248, and the comprehensive index Qi is 0.000. All ranking first, this finding indicates that the public charging infrastructure of this operator has strong competitiveness in the market. In addition, the results are consistent with actual news reports, which also proves the effectiveness of the index system and model.

## 1. Introduction

**Fig 1:**
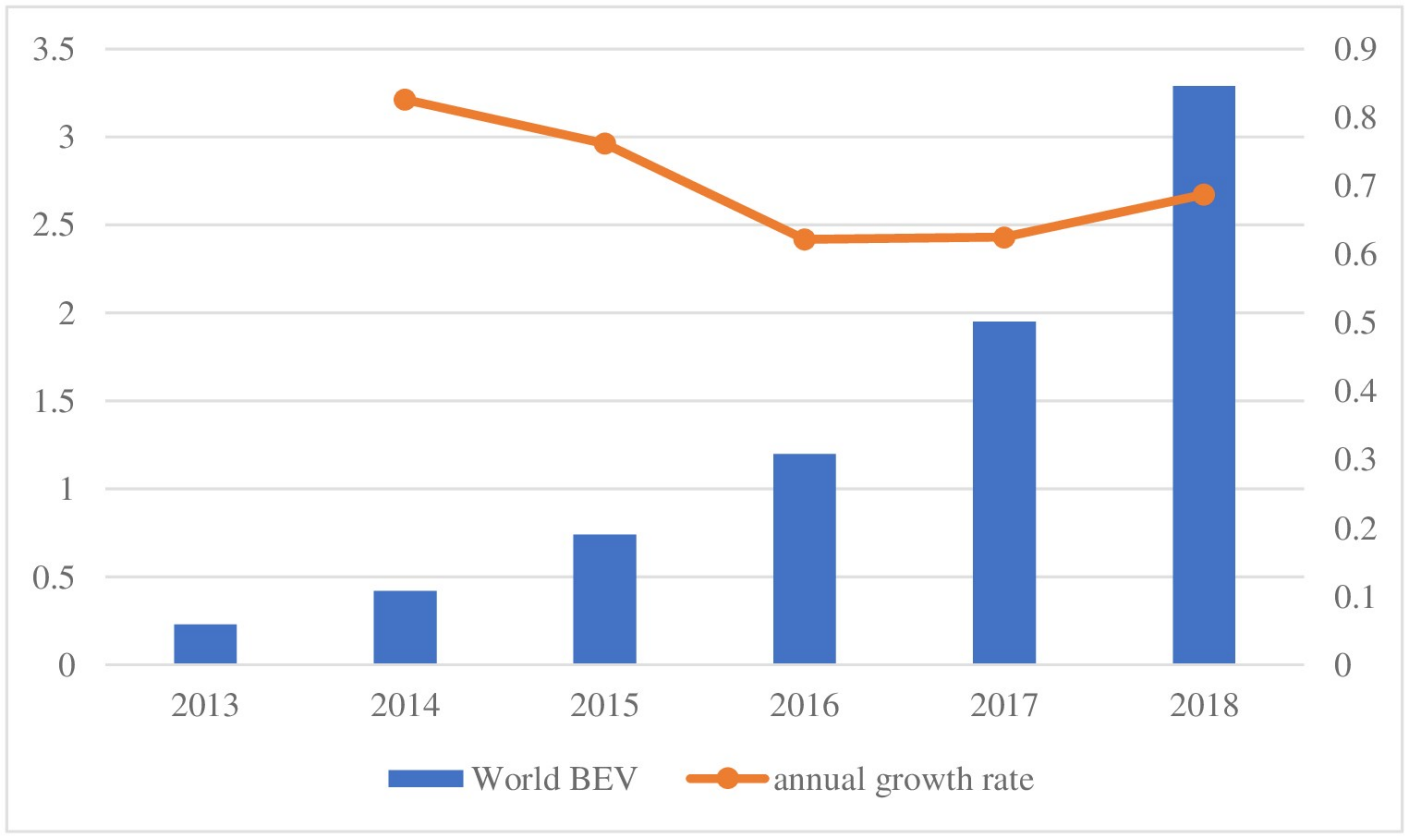
Global number of pure electric vehicles (10,000) and growth rate from 2013 to 2018. The data is from the International Energy Agency (IEA) (2019).

According to statistics, the transportation sector accounts for nearly a quarter of the global gas emissions, and EVs have the advantages of low pollution and low noise compared with traditional fuel vehicles [1]. The United States, Japan, the European Union and other developed countries have promoted the development of new energy vehicles and charging infrastructure to the national strategy level. To ensure the global leading share of the EV market, China introduced a series of guiding policies and invested significant financial expenditures [2–4]. However, China still has the largest EV market, followed by Europe and the United States [5]. The EV30@30 campaign calls on the government to mobilize the private sector, consumers and others to participate in the market with the aim of the sales share of EVs reaching 30% by 2030 [6]. According to statistics from the Global Energy Agency, the number of battery electric vehicles (BEVs) in the world was 230,000 in 2013 and 3.29 million in 2018, an increase of more than 14 times in just five years [5]. Fig 1 shows the global number of pure EVs (10,000 units) and the growth rate from 2013 to 2018. According to the "Global Renewable Energy Outlook: Energy Transformation 2050", the number of EVs in the transportation sector will increase from 8 million in 2019 to 1.1 billion in 2050 [7].

**Fig 2:**
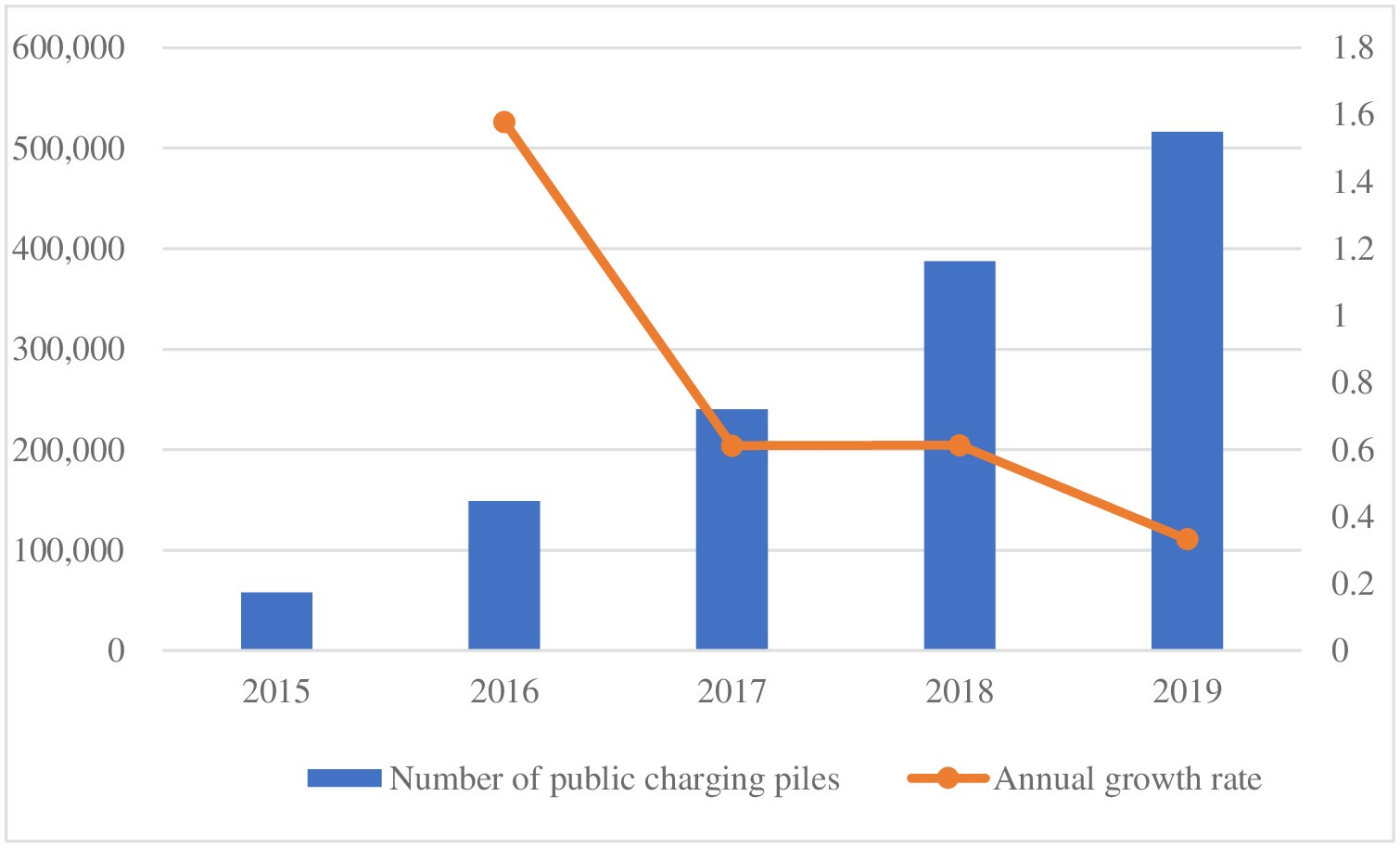
Quarterly amount and annual growth rate of public charging infrastructure in China. The data are from Auto Review (2019). www.autoreview.com.cn.

The development of charging infrastructure will drive the development of the EV industry [8]. Governments of all countries are supporting the deployment of charging infrastructure, and other stakeholders are also increasing their investment in charging infrastructure. Fig 2 shows the quarterly holdings and growth rate of public charging infrastructure in China. Charging infrastructure includes public charging infrastructure, dedicated charging infrastructure and private charging infrastructure. On the premise of having a private charging infrastructure, users are more inclined to charge at home, which can avoid higher charging costs and time costs [9]. However, due to the special national conditions of China’s large population and dense housing, a large number of private charging infrastructure users are diverted from public charging infrastructure users. The main reasons for this phenomenon are as follows: the owner committee does not support the infrastructure, the property service companies do not cooperate, and user-side difficulties in the renovation of power facilities and road pipelines.

The layout of public charging infrastructure for EVs follows the principle of gradually advancing from the urban center to the periphery and from priority development areas to general areas, which can gradually increase the distribution density of public charging infrastructure. According to "the annual Report on the Development of China’s Charging Infrastructure in 2019–2020", the national charging infrastructure in 2019 reached 1.2 million, and the vehicle-to-pile ratio increased from 7.84:1 in 2015 to 3.5:1. The vehicle-to-pile ratio will remain at approximately 3.4:1 in the future [10]. As early as 2018, the utilization rate of public charging facilities in China was still less than 15%, which already showed signs of a relatively high idle rate of some public infrastructure. The consequence of vigorous development will only be that a considerable number of operators will be eliminated, the remaining charging piles will occupy public resources, and the scrapping of charging piles will also cause a waste of resources.

The objective of this paper is to establish an evaluation index system for public charging infrastructure from the perspective of user experience, which can evaluate the existing public charging infrastructure suppliers by using the multi-attribute group decision-making methods. Few existing literatures have established an index system from the perspective of consumer experience. This article can make up for the deficiencies in this field. The combination of subjective and objective methods to rank the three major suppliers can be as close to the actual reality as possible. The example given in this paper confirms the reliability of this method. The proposed indicator system has three contributions: (1) It can be used as a reference for the performance evaluation of public charging infrastructure operators; (2) Formulate incentive policies for public charging infrastructure for the government; (3) Constantly promote the improvement of the public charging infrastructure market.

## 2. Related review

Many studies have focused on the relationship between the amount of charging infrastructure and the increase in EVs, that is, whether the increase in charging infrastructure will promote the development of EVs. Literature [11] established a model of the relationship between German charging infrastructure and the monthly new registrations of EVs. Literature [12] believed that the promotion effect of charging infrastructure on EV sales in emerging markets is more obvious than in mature markets. However, the increase in the number of electric vehicles will also drive new charging infrastructure, which is a mutual influence. In addition, it should not be ignored that the utilization rate of the charging infrastructure should be improved [13,14] in addition to increasing the amount of charging infrastructure.

By understanding the charging behavior habits of EV users and the choice preference of public charging infrastructure operators, public charging infrastructure can be better developed. Literature [15] suggested that women and elderly people are more dependent on family for charging. A long commuting distance is the reason for the hybrid power consumption of PHEVs. Compared with pure EVs, plug-in EVs have shorter battery storage mileage. To reduce fuel consumption, users usually make maximum use of battery storage, so they will mix their use of official charging infrastructure with public charging infrastructure. Literature [9] studied the charging time and location settings of the charging infrastructure under different charging modes (powers) and conducted a literature review of five aspects of the charging infrastructure from the consumer perspective, such as use opportunities, use costs, impact on the power grid and others. The time requirement of public charging infrastructure is generally higher than that of private charging infrastructure because consumers want to minimize the waiting time for charging. Literature [16], proposes that the increase in battery capacity and the improvement of charging power in the future will allow fast charging infrastructure (mostly public charging infrastructure) to achieve the same energy supplement effect as traditional gas stations. The high costs of the development of public charging infrastructure are the challenging target of various stakeholders. Many literatures have quantitatively assessed the number and location of charging piles in order to reduce capital investment [17,18]. However, the above studies mostly focus on the speed, location optimization and quantity control of charging infrastructure. To meet the different needs of consumers, public charging infrastructure usually contains several charging plugs with different powers. Moreover, this paper discusses the improvement of public charging infrastructure services, and the focus of the research is more inclined to consumers’ feelings regarding their use.

The entropy-DEMATEL method is used to weight the established indexes in the public charging infrastructure Multicriteria Decision Making (MCDM) problem. For EV charging infrastructure, [19] used the fuzzy TOPSIS method to discuss the risks of public-private partnership projects of EV charging infrastructure. Although the fuzzy TOPSIS method has similarities with the public charging infrastructure index evaluation system, it only considers the subjective weight of risk and does not consider its objective weight. [20] also studied the risks of electric vehicle charging infrastructure PPP projects, using 2-Tuple and the DEMATEL Model, but this method is not suitable for fuzzy information and incomplete information. [21] used 4 different methods to evaluate 18 public infrastructures of EVs in Lithuania. Although the conclusions obtained are consistent, the calculations are more cumbersome.

For the MCDM approach, literature [22] proposed a data-driven two-stage MCDM framework to investigate the optimal configuration of a stand-alone wind/PV/hydrogen system. Literature [23] proposed a fuzzy MCDM technique based on cumulative prospect theory to select the most appropriate renewable power sources in China. In addition to the application of MCDM in practical problems, research on the improvement of MCDM has also made different amounts of progress. Literature [24] intended to introduce one such information measure defined on Picture Fuzzy Sets in MCDM called R-norm picture fuzzy information measure. Literature [25] introduced a new improved TOPSIS (Technique for Order Preference by Similarity to Ideal Solutions) method based on weighted correlation coefficients.

In recent years, the entropy method has been well applied in environmental assessments [26,27], public infrastructure [28], risk assessment [29], energy [30,31] and other areas. CRITIC [32] focuses more on whether there is a conflict between the indicators and more on the correlation between the indicators. The disadvantage is that the versatility and participability are relatively poor. The weight is determined mainly according to the discreteness of standardized data evaluated by experts, which has strong universality and participation by experts. So the entropy weight method is chosen.

DEMATEL as a weighting method is often applied in combination with game theory [33], fuzzy evidence theory [34], D numbers [35], ANP [36], the grey method [37], etc. In addition, DEMATEL can be applied in supplier selection [33], energy, project investment and other fields. For the DEMATEL approach, literature [38] combined fuzzy theory with the DEMATEL method to discuss the main obstacles in the human resource management of China’s hydrogen refueling stations. The advantage of the DEMATAL method [39] is that it can be combined subjectively and objectively to make a comprehensive assessment, while the other three methods, LBWA [40], FUCOM [41] or BWM [42] models, cannot achieve. However, the comprehensive evaluation of EV charging pile operators in this paper often requires subjective policy preference judgment based on the Chinese government’s policies on the development of electric vehicles.

The advantage of the VIKOR method [43] is that when the units corresponding to the same attribute are different, the final sorting result will not be affected. However, EDAS [44,45], multi-attributive border approximation area comparison (MABAC) [46,47], MAIRCA [48] and CODAS [49], combined compromise solution (CoCoSo) [50], IRN Dombi-Bonferroni (IRNDBM) [51]methods cannot solve the conflict among index attributes, and when the same attribute corresponds to different units, different results will be obtained, which will affect the final ranking result. So choose the VIKOR method. The classification of the literature review is shown in Table 1 below.

**Table 1:** The classification of the literature review.

Number	Content	Literature number
1	The relationship between the amount of charging infrastructure and EVs	[11–14]
2	The charging behavior habits of EV users and the choice preference of public charging infrastructure operators	[9,15–18]
3	EV charging infrastructure assessment	[19–21]
4	Application of MCDM method in energy field	[22–25]
5	Comparison and analysis of Entropy method and other methods	[26–32]
6	Comparison and analysis of DEMATEL method and other methods	[33–42]
7	Comparison and analysis of VIKOR method and other methods	[43–53]

This paper has the following innovations: (a) The public charging infrastructure evaluation index system is established from the EV user perspective. (b) The combination of the objective entropy weight method and subjective DEMATEL method is used to assign weights. The objective entropy weight method can show the objective evaluation of EV owners of the indicators but ignores the subjective factors of human beings. The subjective DEMATEL method can compensate for this deficiency and has the advantage of considering the interaction between indicators (that is, any two indicators at the same level are interrelated). (c) Based on the integration of the above two weighting methods, a VIKOR method, which fully considers the maximization of group benefits and the minimization of individual regret, is applied to rank the three alternative public charging infrastructures; and the decision result is more reasonable.

The following chapters are arranged as follows: Section 2 establishes the evaluation index system of public charging infrastructure from the EV user perspective. Section 3 describes the solution steps of the entropy-DEMATEL-VIKOR model. Section 4 presents the case solutions and analysis of three alternative public charging infrastructures. Finally, a summary of the paper is presented in Section 5.

## 3 Public charging infrastructure evaluation index system

**Fig 3:**
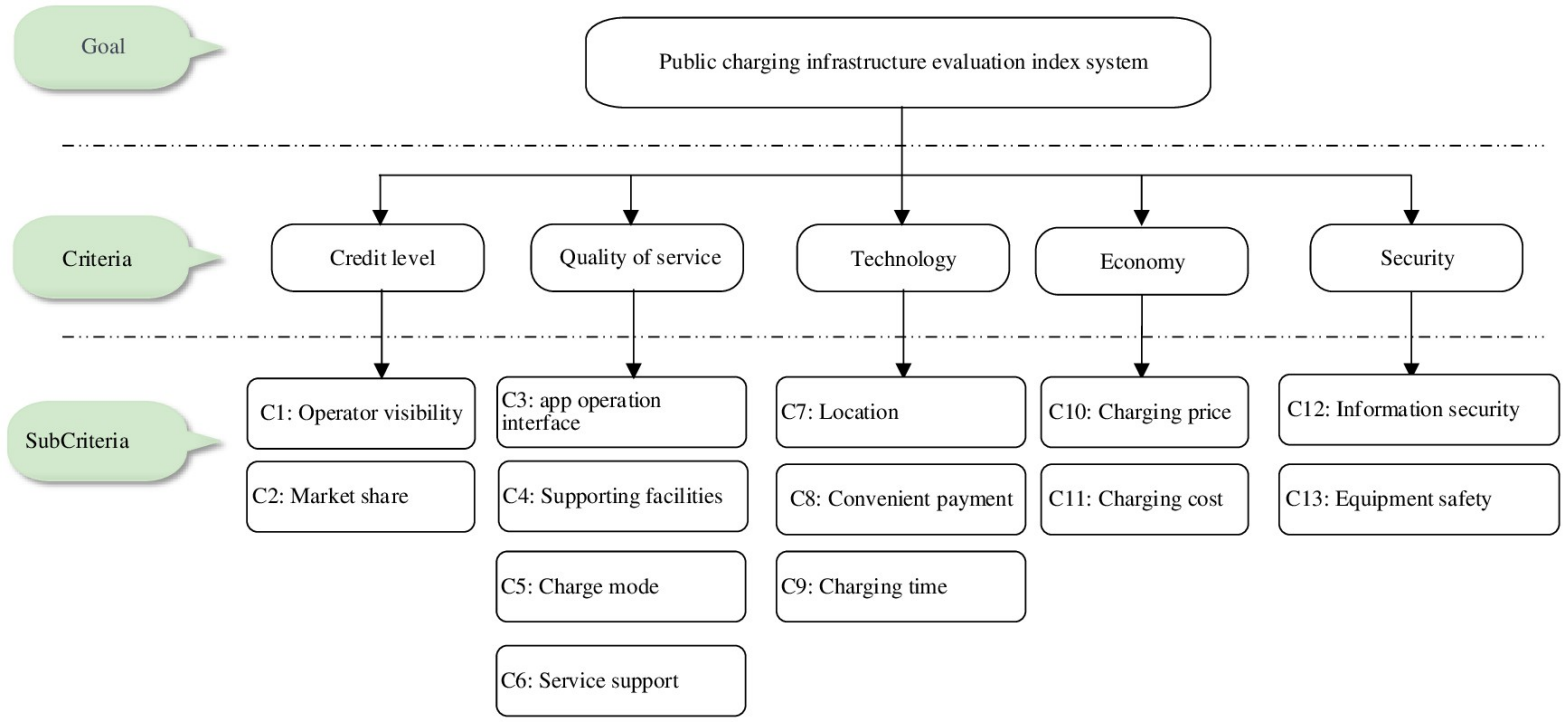
Evaluation index system of public charging infrastructure.

To improve the development of public charging infrastructure operators and provide better services to improve user satisfaction, this paper establishes an evaluation index system of public charging infrastructure from the perspective of consumers with 5 first-level indicators and 13 second-level indicators, as shown in Fig 3. The index system can serve as a reference for the government to formulate incentive policies for the industry.

### 3.1 Credit level

This indicator is often used in the selection of suppliers by enterprises in the field of business administration. The indicator of reputation level in this paper includes two secondary indicators, namely, operator popularity (C_1_) and market share (C_2_). The two indicators (C_1_ and C_2_) are complementary to each other. Operators must have a sufficient market share to improve their brand awareness, and the improvement of their market share will also promote the brand awareness of operators [52]. Consumers are more likely to trust high-profile operators, such as the State Grid’s e-charging. Due to the background of state-owned enterprises, household electricity for consumers and their relatives is also provided by the State Grid, and thus, customers are more willing to trust e-charging provided by the State Grid rather than that provided by other private operators.

### 3.2 Quality of service

Service quality refers to the degree of service that a service provider can provide to consumers. When the degree of service provided by a service provider is greater than the psychological expectation of consumers, the service quality will be higher. However, when the service level provided by a service provider cannot meet the psychological expectations of consumers, the service quality will be lower. To meet the charging needs of EV users, public charging pile operators should provide high-quality services. The first-level index includes four evaluation aspects: the app operating interface (C_3_), supporting facilities (C_4_), charging mode (C_5_) and service support (C_6_).

The app operating interface (C_3_): The app operating interface should be simple and easy to operate [53]. Especially when EVs are in urgent need of charging, most users hope to download the app in the shortest time and achieve charging in the shortest time through operating tips. In addition, the app should contain a comprehensive amount of information, bring accurate information to users, and reduce or avoid the situation of software jams.

Supporting facilities (C_4_): In addition to charging functions, public charging infrastructure should be equipped with public toilets, small shopping supermarkets and other functions to provide customers with more comprehensive services. Supporting facilities can meet various physiological needs of consumers while charging so that the charging process is no longer a boring waiting process.

Charging mode (C_5_): The charging mode includes the amount and proportion of fast and slow charging and the diversity of EV charging plug interfaces.
Service support (C_6_): Service support includes instructions on the steps before charging, timely responses to problems during charging, service feedback after charging, and auxiliary charging for special users (the disabled).

### 3.3 Technology

Advanced technology can provide consumers with better service experiences. Technology can be divided into three aspects: location, convenient payment and charging time.

Location (C_7_): The location of the charging pile is accurately positioned, and the location is convenient [52].
Convenient payment (C_8_): A charging pile should provide a variety of common payment methods and charge detail inquiries.
Charging time (C_9_): The charging time is the waiting time for charging in a queue. This indicator is mostly related to the number of people charging, the number of charging piles, and the set ratio of fast and slow charging. To reduce charging anxiety and obtain the same short “refueling” time as traditional fuel cars, rapid charging has been using technical means to shorten charging times. It is believed that the development of the battery industry in the future will solve the above problems.

### 3.4 Economy

Charging price (C_10_): The charging price can be divided into the uniform unit price throughout the day, peak and valley electricity prices, monthly subscriptions, etc.
Charging cost (C_11_): The charging cost includes the time cost of charging and the power consumption formed by distance.

### 3.5 Security

Information security (C_12_): Private information such as charging information and payment information is safe and not leaked.
Equipment safety (C_13_): Equipment safety ensures the safety of charging, plugging in the charging gun and passing the authentication before power on; setting the emergency power off button; etc.

## 4. Methodology

**Fig 4:**
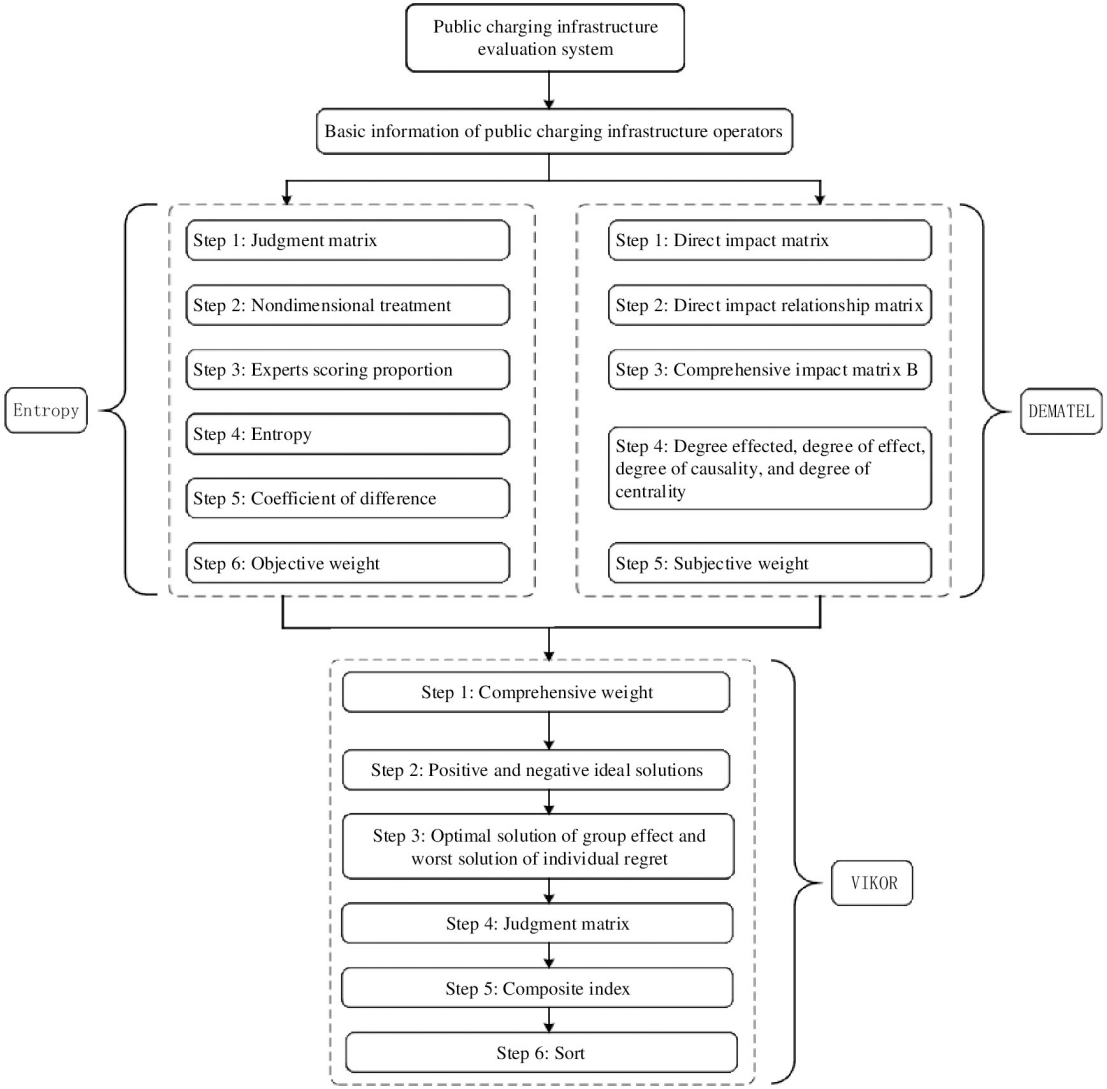
The steps of the comprehensive weighting method.

The objective weighting method adopts the entropy weighting method to determine the weight of each indicator based on the entropy value that can reflect the amount of information in each indicator. However, this method has the disadvantage of not considering the interaction between each indicator [54]. By using graph theory and matrix tools, the subjective weighted DEMATEL method constructs a direct impact matrix that can reflect the logical relationship among various indicators and realize quantitative analysis. Therefore, this article uses a comprehensive weighting method that combines the subjective DEMATEL method and the objective entropy method. The steps of the method are shown in Fig 4, and the weighting and ranking steps are listed in 3.1–3.3.

### 4.1 Entropy method

Entropy [55] is a measure of information uncertainty using probability theory. The more dispersed the data are, the greater the uncertainty of this indicator. Given a scheme and indicators, the entropy weight method can determine the objective weight of each indicator, which is an effective method to determine the weights using information theory [54].

Step 1: Construct the evaluation index judgment matrix. *X* is the evaluation indicator set of evaluation indicators, *m* is the number of questionnaires, and *n* is the number of evaluation indicators. The scores are based on the degree of importance ranging from 1–5; and the higher the score is, the more important the indicator.
$$X=\left(\begin{array}{cccc} x_{11} & x_{12} & \cdots & x_{1n}\\ x_{21} & x_{22} & \cdots & x_{2n}\\ \cdots & \cdots & \cdots & \cdots \\ x_{m1} & x_{m2} & \cdots & x_{mn} \end{array}\right)$$ (1)
Step 2: Conduct the dimensionless processing of data.
$$Y_{ij}=\frac{x_{ij}-\text{min}\left(x_{j}\right)}{\text{max}\left(x_{j}\right)-\text{min}\left(x_{j}\right)}$$ (2)
Step 3: Calculate the scoring proportion *p*_*ij*_ of each expert under the *j*th indicator:
$$p_{ij}=\frac{Y_{ij}}{\sum_{i=1}^{m}Y_{ij}}(i=1,2,\ldots ,m;j=1,2,\ldots ,n)$$ (3)
Step 4: Calculate the entropy value *e*_*j*_ of each indicator:
$$e_{j}=-\frac{1}{\text{In}m}\sum_{i=1}^{m}p_{ij}\text{In}p_{ij}\left(0\leq e_{j}\leq 1\right)(i=1,2,\ldots ,m;j=1,2,\ldots ,n)$$ (4)
Step 5: Calculate the difference coefficient *h*_*j*_ of the indicators:
$$h_{j}=\left|1-e_{j}\right|$$ (5)
Step 6: Calculate the weight $w_{j}^{1}$ of each indicator:
$$w_{j}^{1}=\frac{h_{j}}{\sum_{j=1}^{n}h_{j}}$$ (6)

### 4.2 DEMATEL method

This method uses graph theory and matrix tools to analyze the system indicators. Through the logical relation and the direct impact matrix between each indicator in the system, the degree of influence on other indicators and the degree of influence of other indicators can be calculated to calculate the degree of centrality and degree of causality of each indicator. This method makes full use of the experience and knowledge of experts to address complex problems and is an effective method for indicator analysis and identification [38]. The specific steps are described as follows:

Step 1: Construct the initial direct influence matrix ***C***. *C*_1_, *C*_2_, …, *C*_n_ are evaluation indicators for public charging infrastructure. Evaluation experts use 0, 1, 2, 3, and 4, which represent very low, low, general, high, and very high, respectively, to indicate the degree of influence of index *C*_*i*_ on *C*_*j*_. According to the degree of mutual influence among the public charging infrastructure evaluation indicators scored by the expert team, the influence relationship between indicator *C*_*i*_ and indicator *C*_*j*_ is represented by *C*_*ij*_. Then, the initial direct influence matrix *C* is obtained as follows:
$$C=\left[\begin{array}{cccc} c_{11} & c_{12} & \cdots & c_{1n}\\ \cdots & \cdots & \cdots & \cdots \\ c_{i1} & c_{i2} & \cdots & c_{in}\\ \cdots & \cdots & c_{ij} & \cdots \\ c_{n1} & c_{n2} & \cdots & c_{nn} \end{array}\right](i,j=1,2,\cdots ,n)$$ (7)
  where the elements on the main diagonal are all equal to 0.
Step 2: Standardize the initial direct influence matrix *C* by using formulas (7) and (8) to obtain the direct influence relationship matrix *S*.
$$k=\text{min}\left\{\frac{1}{\underset{1\leq i\leq n}{\text{max}}\sum_{j=1}^{n}\left|c_{ij}\right|},\frac{1}{\underset{1\leq i\leq n}{\text{max}}\sum_{i=1}^{n}\left|c_{ij}\right|}\right\}$$ (8)
$$S=k\times C={\left[s_{ij}\right]}_{n\times n}$$ (9)
Step 3: Obtain the comprehensive impact matrix, denoted as *B*, of the public charging infrastructure evaluation indicators. The comprehensive influence matrix reflects the indirect influencing relationship among the indicators. Formula (9) is used to process the direct influencing relation matrix *S* obtained in step 2 to obtain the comprehensive influence matrix *B*, as shown below:
$$B=S^{1}+S^{2}+S^{3}+\cdots +S^{n}=\sum_{i=1}^{n}S^{i}={\left[b_{ij}\right]}_{n\times n}$$ (10)
  In the formula, *b*_*ij*_ represents the comprehensive degree of influence obtained by indicator *C*_*i*_ from indicator *C*_*j*_. That is, indicator *C*_*i*_ is directly or indirectly affected by indicator *C*_*j*_. In contrast, indicator *C*_*j*_ has a comprehensive influence on indicator *C*_*i*_.
Step 4: Analyze the influence matrix *B* obtained in step 3. First, calculate *b*_*ij*_ to obtain the degree affected *R*_*j*_ and the degree of influence *D*_*i*_ of each index, as shown below:
$$R_{j}=\sum_{i=1}^{n}b_{ij}$$ (11)
$$D_{i}=\sum_{j=1}^{n}b_{ij}$$ (12)
  where the degree influenced *R*_*j*_ represents the sum of the comprehensive influence values of all indicators on indicator *I*, and the degree of influence *D*_*i*_ reflects the combination of the direct and indirect comprehensive influence values of indicator *j* on all other indicators.
  Then, according to the degree influenced *R*_*j*_ and degree of influence *D*_*i*_, the degree of causality *N*_*i*_ and the degree of centrality *M*_*i*_ can be obtained. The formulas are as follows:
$$N_{i}=R_{j}-D_{i}$$ (13)
$$M_{i}=D_{i}+R_{j}$$ (14)
  Among the measures, the degree of causality *N*_*i*_ reflects the comprehensive degree of influence of index *i* on other indicators. When *N*_*i*_ > 0, it indicates that the degree of influence of index *i* on all other indexes is greater than the degree of index *i* affected by other indexes, indicating that the index actively affects other indicators. In contrast, when *N*_*i*_ < 0, the comprehensive influence of other indicators on this index is stronger than its own influence on other indicators, indicating that this index is passively affected. The greater the absolute value of *N*_*i*_ is, the greater the degree of influence. The degree of centrality *M*_*i*_ is the sum of the degree of influence and the degree affected, which reflects the role played by the indicator in the indicator system, that is, the position occupied by the indicator in the whole indicator system. The greater the value of the degree of centrality *M*_*i*_ is, the greater the role that this indicator plays, and the stronger the correlation between this indicator and other indicators in the indicator system.
Step 5: Calculate the weight of each indicator in the evaluation indicator system, which is represented by vector matrix *W*_2_. The greater the weight of the evaluation indicator is, the more important the role the indicator plays in the evaluation process. The evaluation indicator weight is calculated based on the degree of causality *N*_*i*_ and degree of centrality *M*_*i*_ obtained in the previous step. The specific formula is as follows:
$$w_{i}^{\prime }={\left[{\left(M_{i}\right)}^{2}+{\left(N_{i}\right)}^{2}\right]}^{1/2}$$ (15)
$$W_{2}=w_{i}^{2}=\frac{w_{i}^{\prime }}{{\sum_{i=1}^{n}\left[{\left(M_{i}\right)}^{2}+{\left(N_{i}\right)}^{2}\right]}^{1/2}}$$ (16)
Step 6: Calculate the comprehensive weight. To simplify the calculation, the comprehensive weight is calculated using the objective weight (obtained by function 6) and the subjective weight (obtained by function 16) at a ratio of 1:1.
$$W=0.5W_{1}+0.5W_{2}$$ (17)

### 4.3 VIKOR method

Based on the L_P_-metric aggregation function, the compromise solution of each alternative is calculated by determining the “positive ideal solution” and “negative ideal solution”. This method not only considers the subjective preferences of decision makers, but it also fully considers the maximization of the group benefit and the minimization of individual regret.

Step 1: Construct a standardized decision matrix *G*. There are *m* public charging infrastructure operators *h*_1_, *h*_2_, *h*_3_, ⋯, *h*_*m*_ participating in the selection to form an original evaluation matrix *H* = [*h*_*ij*_]_*m*×*n*_, where *h*_*ij*_ represents the initial evaluation value of the public charging infrastructure operator *h*_*i*_ under the evaluation index *h*_*j*_. The following formula is applied to standardize the original evaluation matrix *H*, and the standardized decision-making evaluation matrix *G* can be obtained as follows:
$$g_{ij}=\frac{h_{ij}}{\sqrt{{\sum_{i=1}^{m}\left(h_{ij}\right)}^{2}}}(1\leq i\leq m,1\leq j\leq n)$$ (18)
$$G={\left[g_{ij}\right]}_{m\times n}$$ (19)
Step 2: According to the standardized decision matrix *G*, the negative ideal solution *g*_*i*_^*-*^ and the positive ideal solution *g*_*i*_^*+*^ can be calculated. The calculation formulas are described as follows:
$$g_{i}^{-}=\left[g_{1}^{-},g_{2}^{-},\cdots ,g_{n}^{-}\right]=\left\{\begin{array}{l} \underset{1\leq i\leq m}{\text{max}}\;g_{ij}\\ \underset{1\leq i\leq m}{\text{min}}\;g_{ij} \end{array}\right.$$ (20)
$$g_{i}^{+}=\left[g_{1}^{+},g_{2}^{+},\cdots ,g_{n}^{+}\right]=\left\{\begin{array}{l} \underset{1\leq i\leq m}{\text{max}}g_{ij}\\ \underset{1\leq i\leq m}{\text{min}}g_{ij} \end{array}\right.$$ (21)
Step 3: Calculate the individually regretted worst solution *R*_*i*_ and the group benefit optimal solution *S*_*i*_ of the public charging infrastructure operator.
$$R_{i}=\text{max}\left[w_{j}\left(\left(g_{i}^{+}-g_{ij}\right)/\left(g_{i}^{+}-g_{i}^{-}\right)\right)\right]$$ (22)
$$S_{i}=\sum_{j=1}^{n}w_{j}\left(\left(g_{i}^{+}-g_{ij}\right)/\left(g_{i}^{+}-g_{i}^{-}\right)\right)$$ (23)
Step 4: Calculate the comprehensive index of each public charging infrastructure operator: VIKOR comprehensive index *Q*_*i*_.
$$Q_{i}=\frac{v\left(S_{i}-S^{+}\right)}{\left(S^{-}-S^{+}\right)}+\frac{(1-v)\left(R_{i}-R^{+}\right)}{\left(R^{-}-R^{+}\right)}$$ (24)
  where *R*^+^ = min *R*_*i*_, *R*^‒^ = max *R*_*i*_, *S*^+^ = min *S*_*i*_, and *S*^‒^ = min *S*_*i*_. *R*^+^ represents the smallest individual regret, and *S*^+^ represents the largest group benefit. *Q*_*i*_ represents the comprehensive evaluation index of alternative operators that considers individual regrets and group effects. The aspect that is considered more can be adjusted by the weight *v*. *v* represents the weight to consider the group effect, and (1-*v*) is the weight to consider individual regrets. When *v* = 0.5, the two aspects are considered equally, which means that the decision maker is subjectively eclectic.
Step 5: Rank the operators of public charging infrastructure and determine the operators who are close to the compromise ideal solution. The three comprehensive evaluation values (*S*_*i*,_ *Q*_*i*,_ and *R*_*i*_) of each operator calculated above are ranked, respectively. The smaller the comprehensive evaluation value is, the more obvious the advantages of public charging infrastructure operators.

## 5. Case study

### 5.1 Background

**Fig 5:**
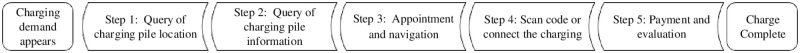
The path diagram for the owner to find the location of the charging pile.

The above methods are used to find the optimal public charging infrastructure operators and find the key points to improve the development of operators. In this case, three different public charging infrastructure operators h1, h2, and h3 in Ji’nan city were selected as the research objects, and 10 EV owners were randomly investigated. Table 2 shows the basic information of the EV owners under investigation. Fig 5 summarizes the path diagram for an owner to find the location of a charging pile. Table 3 summarizes the basic situation of the three public charging infrastructure operators.

**Table 2:** Basic information of EV owners.

Number	Basic information	Classification	Number of people
1	Gender	Male	7
		Female	3
2	Age	18–30	4
		30–45	5
		45 and above	1
3	Monthly income	RMB 50,000–10,000	6
		RMB 10,000–20,000	4
4	Educational background	Junior College and Below	2
		Bachelor degree	6
		Master and above	2

**Table 3:** Basic information of public charging infrastructure operators.

Indicator	Operator h1	Operator h2	Operator h3
Enterprise background	State-owned enterprise	Private enterprise	Foreign company
Market share	49	41	99
Amount of APP downloads	Low	Particularly high	Low
APP interface	General	Convenient	Simple
Fast charge/slow charge	Fast charge is given priority to	Close to 1:1	Slow charge is given priority to
Number of charging piles at stations	Average 8	Quantitative polarization	Average 6
Location characteristics	The parking lot	Shopping mall	Hotel
Method of payment	Charge card	Multiple payment methods	Top up
Price of charging	Peak valley electricity price (cheap)	Uniform price (medium)	Monthly electricity price (most expensive)

### 5.2 Case study

#### 5.2.1 Objective weight

Step 1: Construct the evaluation index judgment matrix.

This questionnaire was distributed to 10 EV owners. Using Eq (1), the judgment matrix *X* = (*x*_*ij*_)_10×13_ as shown in Table 4 is obtained after summary and sorting.

**Table 4:** Judgment matrix.

*X*	*C*_1_	*C*_2_	*C*_3_	*C*_4_	*C*_5_	*C*_6_	*C*_7_	*C*_8_	*C*_9_	*C*_10_	*C*_11_	*C*_12_	*C*_13_
1	1	4	3	4	2	2	5	4	4	5	2	3	2
2	4	2	2	3	4	2	4	4	5	2	4	5	2
3	3	3	3	3	2	2	3	4	4	3	2	2	4
4	4	2	5	4	3	1	4	4	2	4	2	1	4
5	2	4	1	4	3	4	5	4	3	4	3	3	2
6	3	2	4	4	4	5	4	5	2	5	4	1	1
7	3	2	3	2	5	4	4	4	5	4	5	3	2
8	2	4	1	5	3	3	5	2	5	4	3	3	2
9	4	2	5	4	2	2	4	3	1	5	3	1	1
10	3	3	4	4	2	2	5	3	4	4	3	4	2

Step 2: Conduct the dimensionless processing of data.

For Table 4, the dimensionless matrix is calculated using Eq (2) and the results are shown in Table 5.

**Table 5:** Dimensionless matrix.

*Y*	*C*_1_	*C*_2_	*C*_3_	*C*_4_	*C*_5_	*C*_6_	*C*_7_	*C*_8_	*C*_9_	*C*_10_	*C*_11_	*C*_12_	*C*_13_
1	0.00	1.00	0.50	0.67	0.00	0.25	1.00	0.67	0.75	1.00	0.00	0.50	0.33
2	1.00	0.00	0.25	0.33	0.67	0.25	0.50	0.67	1.00	0.00	0.67	1.00	0.33
3	0.67	0.50	0.50	0.33	0.00	0.25	0.00	0.67	0.75	0.33	0.00	0.25	1.00
4	1.00	0.00	1.00	0.67	0.33	0.00	0.50	0.67	0.25	0.67	0.00	0.00	1.00
5	0.33	1.00	0.00	0.67	0.33	0.75	1.00	0.67	0.50	0.67	0.33	0.50	0.33
6	0.67	0.00	0.75	0.67	0.67	1.00	0.50	1.00	0.25	1.00	0.67	0.00	0.00
7	0.67	0.00	0.50	0.00	1.00	0.75	0.50	0.67	1.00	0.67	1.00	0.50	0.33
8	0.33	1.00	0.00	1.00	0.33	0.50	1.00	0.00	1.00	0.67	0.33	0.50	0.33
9	1.00	0.00	1.00	0.67	0.00	0.25	0.50	0.33	0.00	1.00	0.33	0.00	0.00
10	0.67	0.50	0.75	0.67	0.00	0.25	1.00	0.33	0.75	0.67	0.33	0.75	0.33

Step 3: Calculate the scoring proportion *p*_*ij*_ of each expert under the *j*th indicator.

For Table 5, the scoring proportion is calculated using Eq (3) and the results are shown in Table 6.

**Table 6:** Scoring proportion.

*P*	*C*_1_	*C*_2_	*C*_3_	*C*_4_	*C*_5_	*C*_6_	*C*_7_	*C*_8_	*C*_9_	*C*_10_	*C*_11_	*C*_12_	*C*_13_
1	0.0000	0.2500	0.0952	0.1176	0.0000	0.0588	0.1538	0.1176	0.1200	0.1500	0.0000	0.1250	0.0833
2	0.1579	0.0000	0.0476	0.0588	0.2000	0.0588	0.0769	0.1176	0.1600	0.0000	0.1818	0.2500	0.0833
3	0.1053	0.1250	0.0952	0.0588	0.0000	0.0588	0.0000	0.1176	0.1200	0.0500	0.0000	0.0625	0.2500
4	0.1579	0.0000	0.1905	0.1176	0.1000	0.0000	0.0769	0.1176	0.0400	0.1000	0.0000	0.0000	0.2500
5	0.0526	0.2500	0.0000	0.1176	0.1000	0.1765	0.1538	0.1176	0.0800	0.1000	0.0909	0.1250	0.0833
6	0.1053	0.0000	0.1429	0.1176	0.2000	0.2353	0.0769	0.1765	0.0400	0.1500	0.1818	0.0000	0.0000
7	0.1053	0.0000	0.0952	0.0000	0.3000	0.1765	0.0769	0.1176	0.1600	0.1000	0.2727	0.1250	0.0833
8	0.0526	0.2500	0.0000	0.1765	0.1000	0.1176	0.1538	0.0000	0.1600	0.1000	0.0909	0.1250	0.0833
9	0.1579	0.0000	0.1905	0.1176	0.0000	0.0588	0.0769	0.0588	0.0000	0.1500	0.0909	0.0000	0.0000
10	0.1053	0.1250	0.1429	0.1176	0.0000	0.0588	0.1538	0.0588	0.1200	0.1000	0.0909	0.1875	0.0833

Step 4: Calculate the entropy value *e*_*j*_ of each indicator:

For Table 6, the entropy value is calculated using Eq (4) and the results are shown in Table 7.

**Table 7:** Entropy value.

*E*	*C*_1_	*C*_2_	*C*_3_	*C*_4_	*C*_5_	*C*_6_	*C*_7_	*C*_8_	*C*_9_	*C*_10_	*C*_11_	*C*_12_	*C*_13_
e	0.8313	0.6080	0.6731	0.8382	0.6611	0.7945	0.8337	0.8382	0.8197	0.8401	0.7198	0.7304	0.7546

Step 5: Calculate the difference coefficient *h*_*j*_ of the indicators:

For Table 7, the difference coefficient is calculated using Eq (5) and the results are shown in Table 8.

**Table 8:** Difference coefficient.

*H*	*C*_1_	*C*_2_	*C*_3_	*C*_4_	*C*_5_	*C*_6_	*C*_7_	*C*_8_	*C*_9_	*C*_10_	*C*_11_	*C*_12_	*C*_13_
1-e	0.1687	0.3920	0.3269	0.1618	0.3389	0.2055	0.1663	0.1618	0.1803	0.1599	0.2802	0.2696	0.2454

Step 6: Calculate the weight $w_{j}^{1}$ of each indicator:

For Table 8, the objective weight of each indicator is calculated using Eq (6) and the results are shown in Table 9.

**Table 9:** The objective weight of each indicator.

*W*_1_	*C*_1_	*C*_2_	*C*_3_	*C*_4_	*C*_5_	*C*_6_	*C*_7_	*C*_8_	*C*_9_	*C*_10_	*C*_11_	*C*_12_	*C*_13_
Weight	0.055	0.128	0.107	0.053	0.111	0.067	0.054	0.053	0.059	0.052	0.092	0.088	0.080

The objective weights of the indicators in Table 8 show that location (C_7_) and charging cost (C_10_) are not the primary indicators that users stress. This may be due to the widespread distribution of public charging infrastructure in Ji’nan and the convenient charging locations. Although the charging price is divided into direct payments, monthly subscriptions, and recharges, the price per kilowatt-hour is not much different. Moreover, when a user’s EV runs out of power, he or she will not first consider the price and will worry about whether there is enough power left to reach the charging point (C_11_). Three indicators, market share (C_2_), app operating interface (C_3_), and charging method (C_5_), have high weights. This means that users are more willing to choose public charging infrastructure operators with a high market share, simple and easy-to-learn app interfaces, and a variety of charging methods. In addition, the user information security indicator (C_12_) also accounts for a considerable proportion, indicating that users are paying increasingly more attention to the issue of personal information privacy.

#### 5.2.2 Subjective weight

Step 1: Construct the initial direct influence matrix ***C***.

This questionnaire was distributed to 10 EV owners. Using Eq (7), the direct influence average relationship matrix *C* = (*c*_*ij*_)_13×13_ as shown in Table 10 is obtained after summary and sorting.

**Table 10:** Average relationship matrix.

*C*	*C*_1_	*C*_2_	*C*_3_	*C*_4_	*C*_5_	*C*_6_	*C*_7_	*C*_8_	*C*_9_	*C*_10_	*C*_11_	*C*_12_	*C*_13_
*C*1	0	3.7	0.7	0.4	1.9	1.1	2.5	0.8	3	3.8	2.3	3.3	2.6
*C*2	1.3	0	0.2	2.2	0.7	3.1	2.9	2.7	2.3	3.8	1.3	3.2	3.9
*C*3	2.3	2.3	0	3.6	3	1	0.8	3.9	3.5	1.9	1.8	2.9	1
*C*4	3.9	1.2	1.4	0	3.2	0.1	2.1	1.9	0.6	2.1	1.2	0.2	3.3
*C*5	2.2	1.6	2.9	0.7	0	2.5	3.6	0.9	0.8	3.4	3.6	0.2	3
*C*6	3.6	2.2	2.3	2.5	2.9	0	3.1	0.1	3	3.2	3.5	1.7	2.6
*C*7	3.8	2.4	0.9	2.8	1.1	1.1	0	3.8	0.6	2.6	0.8	3.9	3
*C*8	2.6	3.3	0.5	3.7	2	2.4	3.7	0	3.7	1	2.9	0.7	0.4
*C*9	2.9	0.3	0	1.2	0.5	3.3	1.9	1.5	0	1.9	1	1.9	1.3
*C*10	3.6	3.7	3	3.6	0.2	2	0.3	0.5	3.3	0	2	0.1	3.5
*C*11	2.5	2.6	3.1	2	2.1	2	2.9	3.3	0.5	1.4	0	1.4	3
*C*12	1.9	1.9	0.3	2.1	0.1	2.4	1.1	3.8	0.8	1.3	1.4	0	2.7
*C*13	3.6	3.4	2	1	2	0.7	1.4	3.8	3	2.7	0.8	1.2	0

Step 2: Standardize the initial direct influence matrix *C* by using formulas (7) and (8) to obtain the direct influence relationship matrix *S*, as shown in Table 11.

**Table 11:** Direct influence relationship matrix.

*S*	*C*_1_	*C*_2_	*C*_3_	*C*_4_	*C*_5_	*C*_6_	*C*_7_	*C*_8_	*C*_9_	*C*_10_	*C*_11_	*C*_12_	*C*_13_
*C*1	0.000	0.108	0.020	0.012	0.056	0.032	0.073	0.023	0.088	0.111	0.067	0.096	0.076
*C*2	0.038	0.000	0.006	0.064	0.020	0.091	0.085	0.079	0.067	0.111	0.038	0.094	0.114
*C*3	0.067	0.067	0.000	0.105	0.088	0.029	0.023	0.114	0.102	0.056	0.053	0.085	0.029
*C*4	0.114	0.035	0.041	0.000	0.094	0.003	0.061	0.056	0.018	0.061	0.035	0.006	0.096
*C*5	0.064	0.047	0.085	0.020	0.000	0.073	0.105	0.026	0.023	0.099	0.105	0.006	0.088
*C*6	0.105	0.064	0.067	0.073	0.085	0.000	0.091	0.003	0.088	0.094	0.102	0.050	0.076
*C*7	0.111	0.070	0.000	0.082	0.032	0.032	0.000	0.111	0.018	0.076	0.023	0.114	0.088
*C*8	0.076	0.096	0.015	0.108	0.058	0.070	0.108	0.000	0.108	0.029	0.085	0.020	0.012
*C*9	0.085	0.009	0.026	0.035	0.015	0.096	0.056	0.044	0.000	0.056	0.029	0.056	0.038
*C*10	0.105	0.108	0.088	0.105	0.006	0.058	0.009	0.015	0.096	0.000	0.058	0.003	0.102
*C*11	0.073	0.076	0.091	0.058	0.061	0.058	0.085	0.096	0.015	0.041	0.000	0.041	0.088
*C*12	0.056	0.056	0.009	0.061	0.003	0.070	0.032	0.111	0.023	0.038	0.041	0.000	0.079
*C*13	0.105	0.099	0.058	0.029	0.058	0.020	0.041	0.111	0.088	0.079	0.023	0.035	0.000

Step 3: Obtain the comprehensive impact matrix, denoted as *B*, of the public charging infrastructure evaluation indicators, as shown in Table 12.

**Table 12:** Comprehensive influence matrix.

*B*	*C*_1_	*C*_2_	*C*_3_	*C*_4_	*C*_5_	*C*_6_	*C*_7_	*C*_8_	*C*_9_	*C*_10_	*C*_11_	*C*_12_	*C*_13_
*C*1	0.216	0.288	0.130	0.175	0.169	0.176	0.233	0.196	0.246	0.293	0.203	0.228	0.271
*C*2	0.272	0.201	0.123	0.235	0.150	0.232	0.254	0.253	0.240	0.301	0.186	0.229	0.313
*C*3	0.291	0.258	0.115	0.269	0.214	0.180	0.205	0.282	0.268	0.250	0.203	0.219	0.233
*C*4	0.288	0.197	0.133	0.132	0.193	0.119	0.200	0.194	0.159	0.221	0.155	0.122	0.254
*C*5	0.284	0.241	0.197	0.189	0.129	0.207	0.266	0.201	0.194	0.286	0.243	0.149	0.282
*C*6	0.354	0.282	0.197	0.256	0.226	0.163	0.279	0.205	0.273	0.312	0.261	0.210	0.305
*C*7	0.318	0.258	0.106	0.237	0.153	0.169	0.167	0.271	0.184	0.259	0.165	0.240	0.277
*C*8	0.297	0.279	0.126	0.265	0.185	0.211	0.279	0.173	0.264	0.227	0.226	0.166	0.219
*C*9	0.242	0.151	0.107	0.154	0.108	0.192	0.176	0.163	0.124	0.193	0.136	0.155	0.181
*C*10	0.314	0.286	0.192	0.256	0.136	0.192	0.176	0.184	0.260	0.193	0.194	0.144	0.290
*C*11	0.298	0.273	0.199	0.229	0.194	0.200	0.259	0.273	0.193	0.240	0.152	0.186	0.286
*C*12	0.225	0.204	0.094	0.187	0.105	0.175	0.167	0.235	0.157	0.184	0.152	0.106	0.225
*C*13	0.310	0.280	0.161	0.191	0.176	0.164	0.208	0.268	0.252	0.263	0.166	0.172	0.191

**Fig 6:**
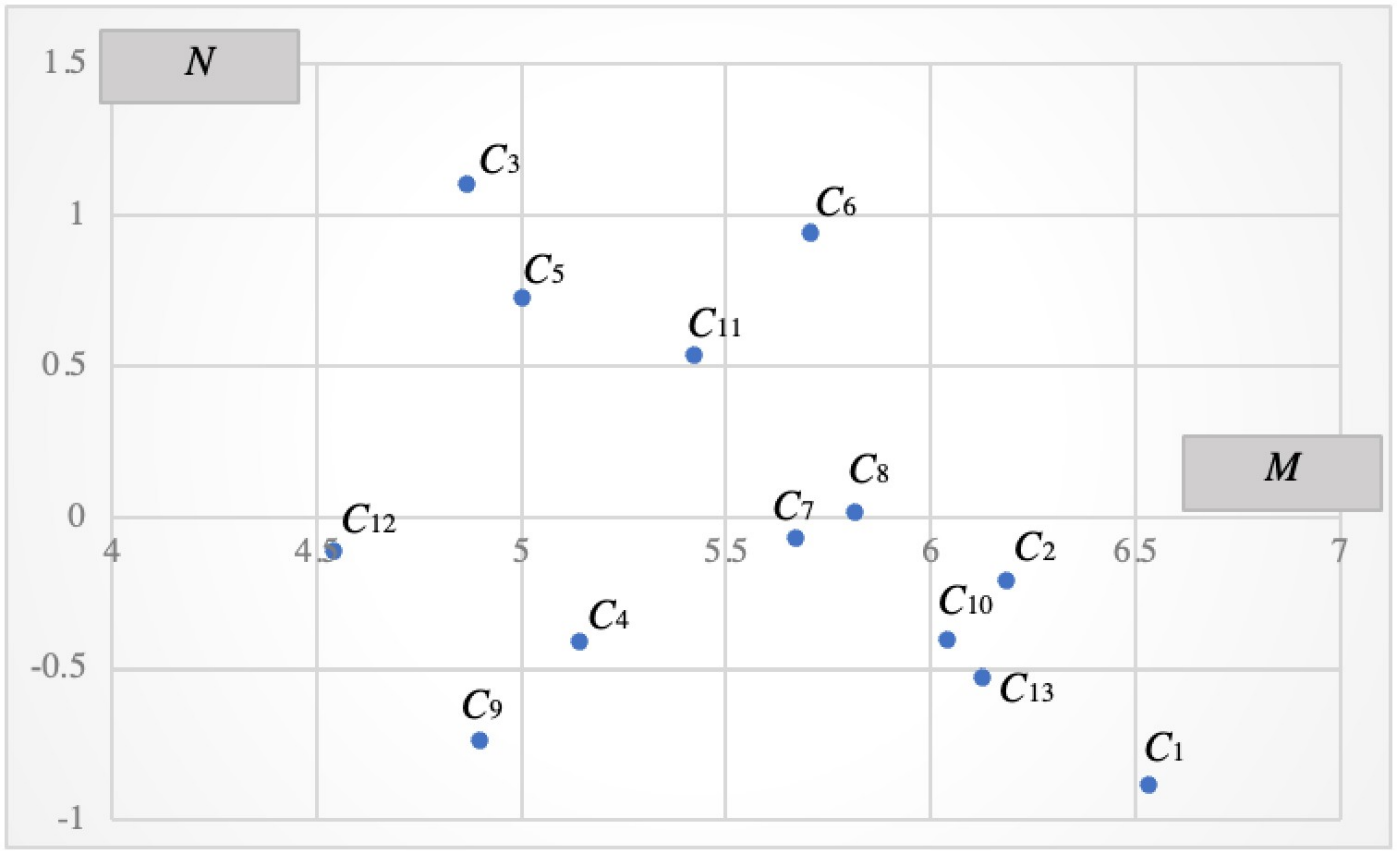
The cause-effect diagram of all indicators.

Step 4: Analyze the influence matrix *B* and Step 5: Calculate the weight of each indicator in the evaluation indicator system, which is represented by vector matrix *W*_2_. Table 13 is the subjective weight results obtained by the Dematel method and Fig 6 is the causal diagram of all indicators.

**Table 13:** Results of DEMATEL method.

Indicators	Influenced degree *R*_*i*_	Influence degree *D*_*j*_	Cause degree *N*_*i*_	Centrality degree *M*_*i*_	Weight *W*_2_
*C*1	3.708	2.823	-0.885	6.531	0.091
*C*2	3.198	2.989	-0.209	6.186	0.086
*C*3	1.880	2.986	1.106	4.867	0.069
*C*4	2.774	2.366	-0.408	5.140	0.071
*C*5	2.136	2.867	0.731	5.003	0.070
*C*6	2.381	3.324	0.943	5.704	0.080
*C*7	2.868	2.802	-0.066	5.670	0.078
*C*8	2.898	2.917	0.019	5.815	0.080
*C*9	2.815	2.080	-0.735	4.896	0.068
*C*10	3.221	2.818	-0.403	6.039	0.084
*C*11	2.441	2.982	0.541	5.422	0.075
*C*12	2.325	2.217	-0.109	4.542	0.063
*C*13	3.325	2.799	-0.526	6.124	0.085

Step 6: Calculate the comprehensive weight. Using Eq (17), the comprehensive weight values are obtained, as shown in Table 14.

**Table 14:** Comprehensive weight values.

indicators	*W*_1_	*W*_2_	Comprehensive weight W
*C*1	0.055	0.091	0.073
*C*2	0.128	0.086	0.107
*C*3	0.107	0.069	0.088
*C*4	0.053	0.071	0.062
*C*5	0.111	0.070	0.090
*C*6	0.067	0.080	0.074
*C*7	0.054	0.078	0.066
*C*8	0.053	0.080	0.067
*C*9	0.059	0.068	0.064
*C*10	0.052	0.084	0.068
*C*11	0.092	0.075	0.084
*C*12	0.088	0.063	0.076
*C*13	0.080	0.085	0.083

The weights obtained by the objective entropy weight method and the weights obtained by the subjective DEMATEL method are different in the ranking of the indicators. This is because the weights obtained by the entropy weight method are ranked according to the importance of the indicators in the public charging infrastructure indicator system. However, the weights obtained by the DEMATEL method are obtained on the basis of calculating the degree of influence and the degree influenced of each index, which reflects the mutual relationship among each indicator. In short, the indicator results obtained by the DEMATEL method have little difference.

The weight of the operator popularity (C_1_) indicator ranks first. This result is because well-known operators are more likely to be trusted by EV users. In addition, high popularity represents a higher market share, perfect facilities, good service, more competitive prices, superior facility locations, etc. Therefore, EV users are more likely to choose a "familiar" charging operator when charging for the first time.

**Fig 7:**
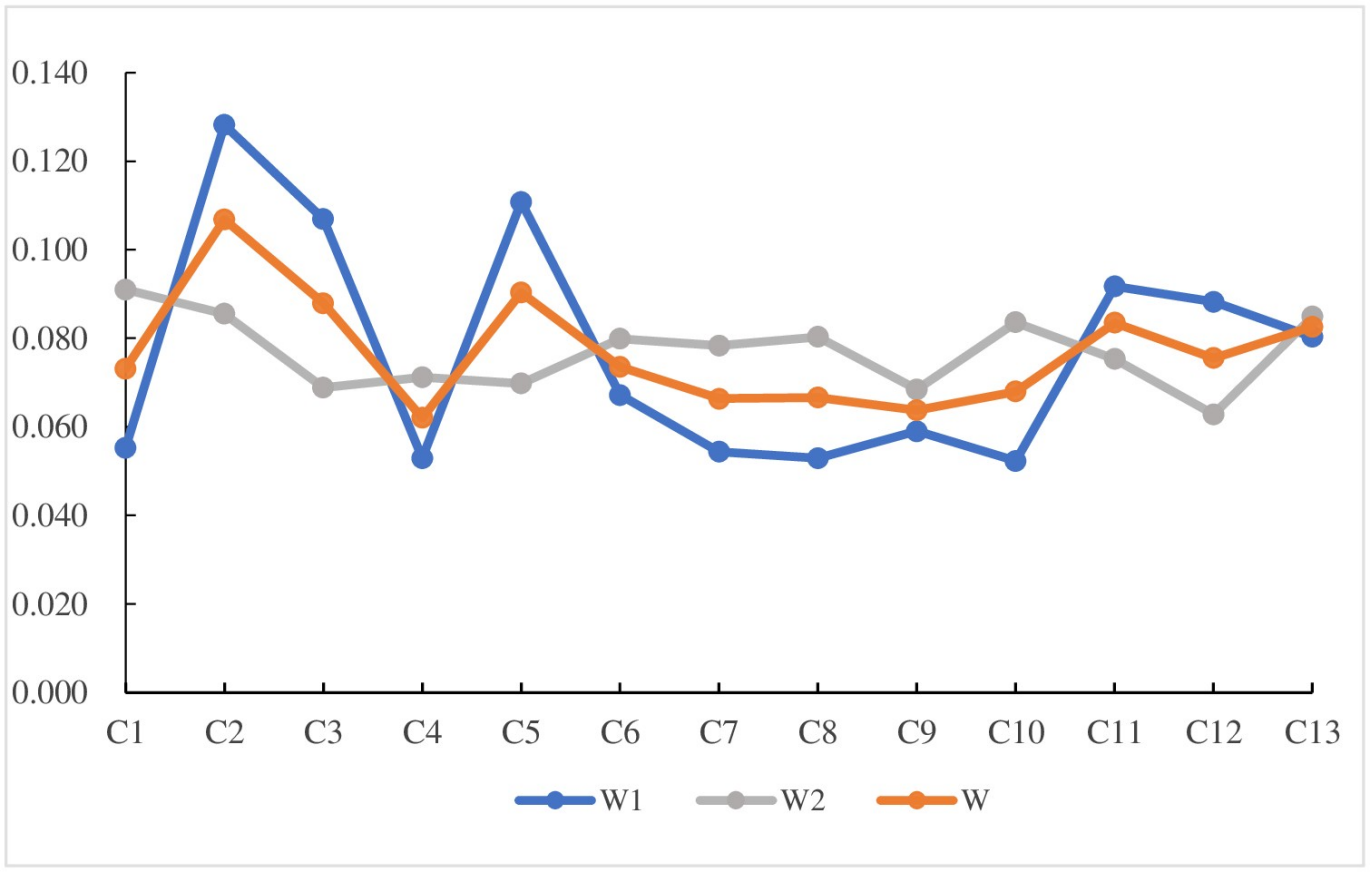
Subjective, objective and comprehensive weight.

Table 14 shows the final comprehensive weight table, and Fig 7 shows the subjective, objective and comprehensive weight charts. The figures show that the subjective weights obtained by the DEMATEL method have little difference, and the indicators are all influenced by each other. The trend of the comprehensive weight W is basically consistent with the trend of objective weight W1. The top three indicators of this survey result are market share C_2_, app operating interface C_3_ and charging method C_5_.

#### 5.2.3 Ranking

Step 1: Construct a standardized decision matrix *G*.
  Taking the public charging infrastructure of Ji’nan city as the research background, three different charging operators *h*_1_, *h*_2_ and *h*_3_ were randomly selected. According to the actual investigation situation, the fuzzy language (poor, general, good, good) = (0.3, 0.5, 0.7, 0.9) was used for numerical processing, and the normalized decision matrix *G* was obtained by Eqs (18) and (19).
$$G=\left[\begin{array}{lllllllllllll} 0.72 & 0.56 & 0.55 & 0.58 & 0.55 & 0.70 & 0.63 & 0.59 & 0.63 & 0.58 & 0.50 & 0.58 & 0.58\\ 0.56 & 0.40 & 0.77 & 0.58 & 0.77 & 0.50 & 0.45 & 0.76 & 0.63 & 0.58 & 0.70 & 0.58 & 0.58\\ 0.40 & 0.72 & 0.33 & 0.58 & 0.33 & 0.50 & 0.63 & 0.25 & 0.45 & 0.58 & 0.50 & 0.58 & 0.58 \end{array}\right]$$
Step 2: According to the standardized decision matrix *G*, the negative ideal solution *g*_*i*_^*-*^ and the positive ideal solution *g*_*i*_^*+*^ can be calculated. Using Eqs (20) and (21), the positive and negative ideal solutions of each operator are determined, and the results are shown in Table 15.
Step 3: Calculate the individually regretted worst solution *R*_*i*_ and the group benefit optimal solution *S*_*i*_ of the public charging infrastructure operator.
Step 4: Calculate the comprehensive index of each public charging infrastructure operator: VIKOR comprehensive index *Q*_*i*_.
Step 5: Rank the operators of public charging infrastructure and determine the operators who are close to the compromise ideal solution.

**Table 15:** The positive and negative ideal solutions of each operator.

Operator	*C*_1_	*C*_2_	*C*_3_	*C*_4_	*C*_5_	*C*_6_	*C*_7_	*C*_8_	*C*_9_	*C*_10_
$g_{i}^{+}$	0.72	0.72	0.77	0.58	0.77	0.70	0.63	0.76	0.63	0.58
$g_{i}^{-}$	0.40	0.40	0.33	0.58	0.33	0.50	0.45	0.25	0.45	0.58
Operator	*C*_11_	*C*_12_	*C*_13_							
$g_{i}^{+}$	0.70	0.58	0.58							
$g_{i}^{-}$	0.50	0.58	0.58							

According to the positive and negative ideal solutions of each operator, the relative degree of closeness *S*_*i*_ of each operator to the positive ideal solution and the relative degree of distance *R*_*i*_ of the negative ideal solution were calculated by Eqs (22)–(24). The comprehensive index *Q*_*i*_ of each operator is calculated to select the best compromised operator. The results are shown in Table 16.

**Table 16:** Operator ranking (V = 0.5).

Operator	Positive ideal solution *S*_*i*_		Negative ideal solution *R*_*i*_		Comprehensive index *Q*_*i*_	
	Value	Rank	Value	Rank	Value	Rank
*h*_1_	0.084	1	0.248	1	0.000	1
*h*_2_	0.107	3	0.284	2	0.561	2
*h*_3_	0.090	2	0.539	3	0.645	3

### 5.3 Sensitivity analysis

**Fig 8:**
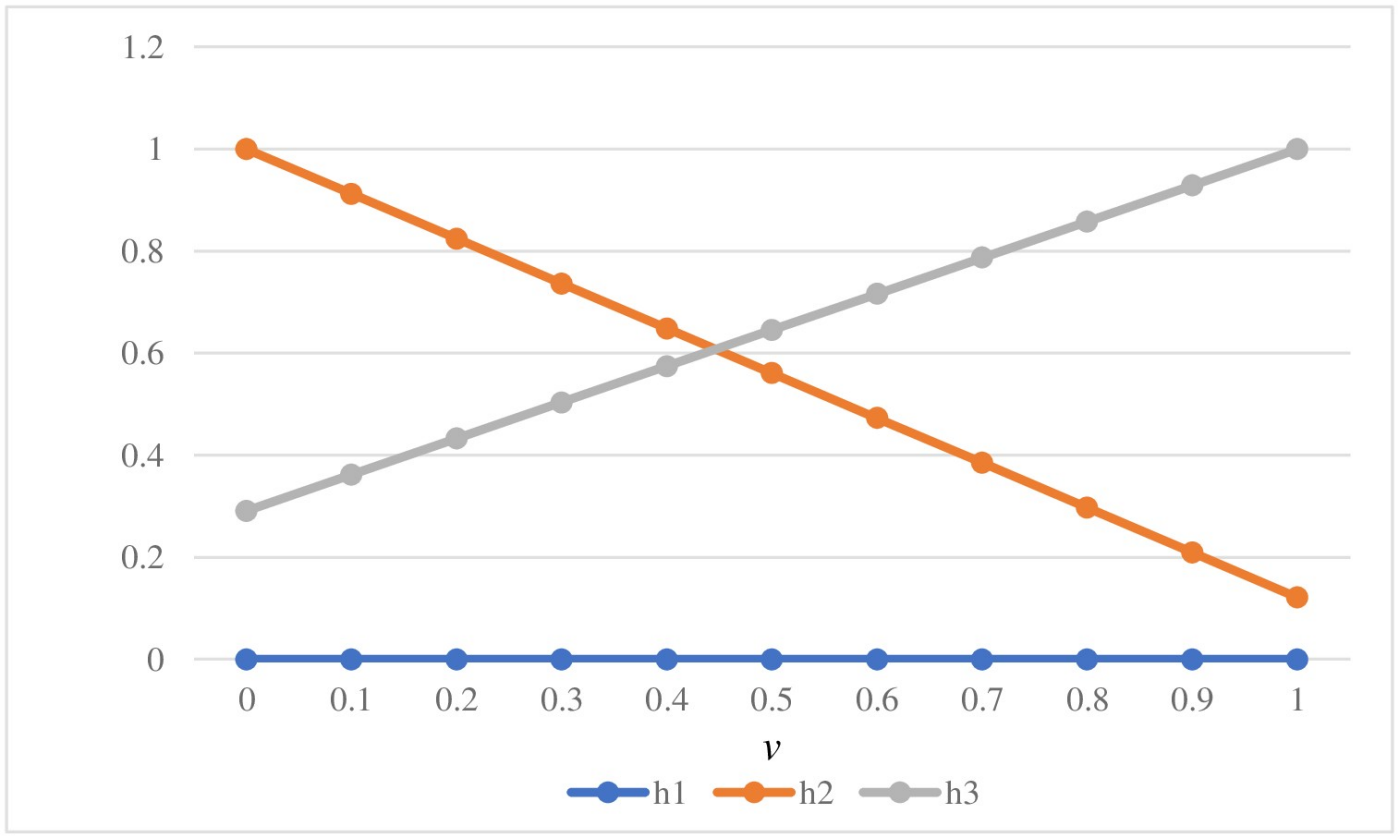
The ranking order trend.

In the proposed VIKOR model, parameter *v* plays an indispensable role and is usually valued at 0.5. However, in fact, *v* can be assigned any value between 0 and 1. Since the decisions made by EV owners will directly lead to different final orderings of public charging infrastructure, it is necessary to change the value of parameter *v* for sensitivity analysis to test the validity and robustness of the results obtained in this paper. Based on the various values of v from 0 to 1, the values of *Qi* are calculated using Eq (24) in Table 17, and the ranking order trend is vividly demonstrated in Fig 8. As seen in Fig 8, the ranking order trends of the three alternatives are not at all influenced by the value of *v* and the comprehensive index *Qi* of *h*_1_ is always ranked first. This result shows that the acquired evaluation results of the proposed entropy-DEMATEL-VIKOR model are robust and reliable.

**Table 17:** The values of *Qi* based on the various values of *v* from 0 to 1.

*v*	*h*_1_	*h*_2_	*h*_3_
0	0.000	1.000	0.291
0.1	0.000	0.912	0.362
0.2	0.000	0.824	0.432
0.3	0.000	0.736	0.503
0.4	0.000	0.648	0.574
0.5	0.000	0.561	0.645
0.6	0.000	0.473	0.716
0.7	0.000	0.385	0.787
0.8	0.000	0.297	0.858
0.9	0.000	0.209	0.929
1	0.000	0.121	1.000

### 5.4 Computation of ranking stability based on different MCDM methods

**Fig 9:**
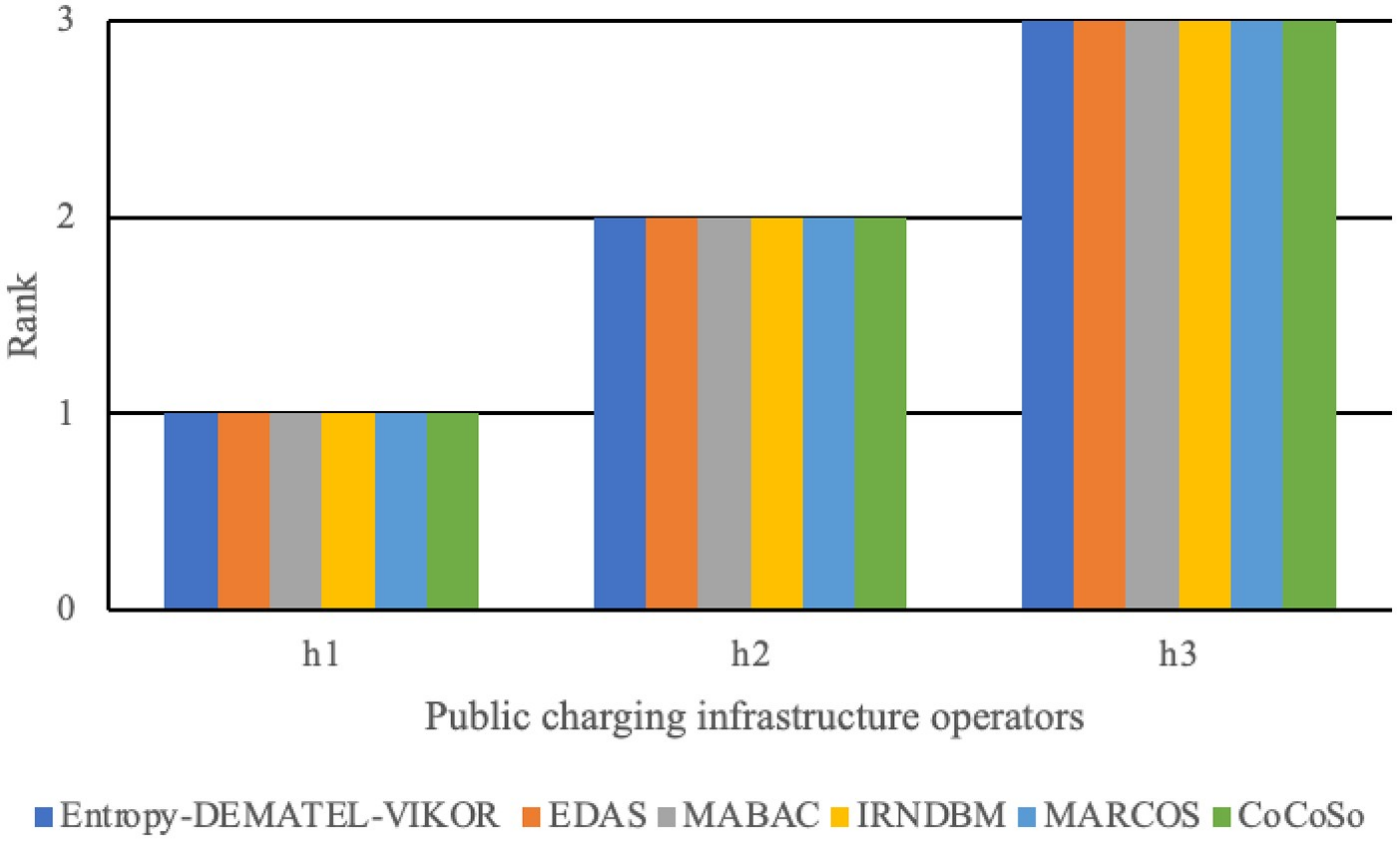
Comparison of the ranking orders.

In order to make a final selection of the best operator of public charging infrastructure, a comparison of the ranking results with those obtained from the EDAS [45,56], MABAC [47,57], IRNDBM [51], MARCOS [58] and CoCoSo [50,59] methods is shown in Fig 9. According to this comparative analysis, the ranking orders of the considered operator of public charging infrastructure obtained by the proposed model are almost similar to those derived by the EDAS, MABAC and CoCoSo methods. Thus, the Entropy-DEMATEL-VIKOR model can be employed for rational decision-making for evaluation of the selection of the best operator of public charging infrastructure.

### 5.5 Results and discussion

Table 16 shows that the comprehensive evaluation value of operator h1 is the best, which is a compromise operator closest to the ideal; followed by operators h2 and h3. The positive ideal solution *S*_*i*_, the negative ideal solution *R*_*i*_, and the comprehensive index *Q*_*i*_ of the operator h1 are all ranked first, indicating that h1 has the maximum group benefit and the minimum individual regret; therefore, it is the best operator of public charging infrastructure. In addition, operator h2 performs well in individual regret but insufficiently in group benefit. Operator h2 ranks second comprehensively and can also be used as an alternative. Among the operators, operator h1 is the State Grid, operator h2 is TGood EVC, and operator h3 is EV Power. In recent online news reports, the charging piles of the State Grid have been rated as the most competitive operators in China, which is consistent with the conclusion drawn in this article. This shows that this method, which has certain scientific and practical significance, can be applied to other similar public charging infrastructure selection research.

## 6 Conclusion

The improvement of public charging infrastructure, first, solves the problem of charging difficulty for EV users and, second, can drive the development of the EV industry to achieve the goal of green and low-carbon transportation. This paper investigates the main indicators that affect EV users’ choice of operators and summarizes the key points of operators’ service upgrades. Therefore, a public charging infrastructure evaluation index system consisting of 5 first-level indicators and 13 second-level indicators was established. The objective entropy weighting method and DEMATEL subjective weighting method were combined to weight the indicators, and then the three charging operators in Ji’nan city were ranked by the VIKOR ranking method. This research can serve as a reference for governments to formulate incentive policies to guide the development of public charging infrastructure.

Based on the research results of this paper, the following improvement measures are proposed for operators: (a) Increase the market share of operators since a high market share makes it easier to gain user trust. (b) Improve the application operating interface. The interface should be as simple as possible and easy to learn. (c) According to the user’s needs, the ratio of charging facilities should be reasonably configured. For example, in office buildings and residential areas, the ratio of slow charging interfaces can be increased; and shopping malls can appropriately reduce their number of slow charging interfaces. (d) Pay attention to protecting the privacy of user information. (e) Improve the supporting facilities, such as toilets, convenience stores, etc., around charging facilities.

The results show that due to the small difference in charging prices among charging operators and the high density of charging facilities, the price and location are no longer the attention focus of EV users. The method proposed in this paper can capture the consumption tendency of current users and potential future users as much as possible in a limited time. This finding can improve the public charging infrastructure and increase the speed of promoting the launch of electric vehicles in the market to solve the problem of high carbon emissions by traditional fuel vehicles. In the future, the improvement of public charging infrastructure after the addition of unmanned EVs and the information security of EV users connected to charging facilities under a smart grid can be studied.

## Supporting information

S1 File (XLS)
